# Microbiomic differences at cancer-prone oral mucosa sites with marijuana usage

**DOI:** 10.1038/s41598-019-48768-z

**Published:** 2019-09-03

**Authors:** Taylor Newman, Laya P. Krishnan, Jessica Lee, Guy R. Adami

**Affiliations:** 1 Department of Periodontics, College of Dentistry, University of Illinois at Chicago, 801 South Paulina Street, Chicago, IL USA; 2 Department of Oral Medicine & Diagnostic Sciences, Center for Molecular Biology of Oral Diseases, College of Dentistry, University of Illinois at Chicago, 801 South Paulina Street, Chicago, IL USA

**Keywords:** Oral cancer, Risk factors

## Abstract

Marijuana smoke contains cannabinoids, immunosuppressants, and a mixture of potentially-mutagenic chemicals. In addition to systemic disease, it is thought to contribute to oral disease, such as tooth loss, tissue changes in the gums and throat, and possibly oral pharyngeal cancer. We used a cross-sectional study of 20 marijuana users and 19 control non-users, to determine if chronic inhalation-based exposure to marijuana was associated with a distinct oral microbiota at the two most common sites of head and neck squamous cell carcinoma (HNSCC), the lateral border of the tongue and the oral pharynx. At the tongue site, genera earlier shown to be enriched on HNSCC mucosa, *Capnocytophaga*, *Fusobacterium*, and *Porphyromonas*, were at low levels in marijuana users, while *Rothia*, which is found at depressed levels on HNSCC mucosa, was high. At the oral pharynx site, differences in bacteria were distinct, with higher levels of *Selenomonas* and lower levels of *Streptococcus* which is what is seen in HNSCC. No evidence was seen for a contribution of marijuana product contaminating bacteria to these differences. This study revealed differences in the surface oral mucosal microbiota with frequent smoking of marijuana.

## Introduction

Marijuana is the most commonly used recreational drug in the United States. In 2015, it was reported that 22.2 million U.S. individuals ≥12 years old had used marijuana in the past month. With a shift toward legalization of the drug, questions arise regarding the health implications of marijuana utilization^1^. Besides the well-known cognitive effects of marijuana usage, it is suspected to have a role in a number of other types of conditions based on its component properties. For example, marijuana smoke may have carcinogenic capabilities as it contains potentially carcinogenic aromatic hydrocarbons^2,3^. In addition, marijuana contains cannabinoids which can bind both CB1 and CB2 cannabinoid receptors on immune cells and have been shown to have strong effects on immune cell function and can alter inflammation^4–7^.

The term “cannabis stomatitis” has been used to describe the oral epithelial changes that occur with inhalation and chewing of cannabis^8^. These changes include leukoedema of the oral mucosa, and hyperkeratosis and leukoplakia at various oral sites. In addition, gingival inflammation can occur with chronic use^8–10^. Intraorally, frequent recreational cannabis use, including marijuana and hashish, was associated with higher risk of severe periodontitis, including deeper probing depths and more clinical attachment loss. Even after excluding former and current tobacco users, poor periodontal status was twice as likely in frequent cannabis users versus non-users^11^. Chronic usage is associated with reduced airway function, increased airway infection, and impairment of macrophage activity, though the evidence for the latter effects is limited^7,12,13^. Marijuana usage has been linked to increased incidence of precancerous mucosal histology in both the head and neck and bronchi in some studies^10^. Some epidemiological studies suggest increased risk of head and neck and lung cancer with marijuana usage, however the risk is lower than that with usage of tobacco and the increased risk is currently debatable^12,14–16^. At this point, the evidence for effects of marijuana on systemic disease is poor in part due to limited data on usage as a result of its illegal nature. With legalization, this will change and further research can come about.

Studies regarding gut microbiome, and more recently the oral cavity, have shown linkages between differences in microbiome at these sites and incidence of a number of diseases including cancer, diabetes, and autoimmune disorders. The state of the oral microbiome can cause dental caries and play a role in periodontal disease initiation and progression. Evidence is beginning to accumulate that changes in oral bacteria can be associated with head and neck cancer and other distal cancers^17^. Researchers have noted taxonomic differences in samples collected from lesions in patients with oral squamous cell carcinoma (OSCC) versus those from normal mucosa^18–21^. When compared to healthy controls, OSCC lesions showed increased bacterial diversity and increased relative abundances of certain taxa at the phylum (*Spirochaetes*, *Fusobacteria*, *and Bacteroidetes)* and genus (*Fusobacterium*, *Treponema*, *Dialister*, *Catonella*, *Filifactor*, *Peptococcus*, *Parvimonas*, *Peptostreptococcus*, *Campylobacter*, *and Pseudomonas*) levels^18,21,22^. It is apparent that the oral mucosal microbiome is different in the presence of disease, such as OSCC, and even in precancerous lesions versus healthy tissue. However, a causative role for bacteria in OSCC incidence or progression is speculative^19^. The upper and lower digestive tract microbiome which can be altered due to diet, air quality, or lifestyle^23–25^ may be an important link between environment and disease.

The aim of this study was to evaluate the hypothesis that marijuana use via inhalation is associated with differences in the oral mucosal microbiome compared to nonusers. In this study two major sites of head and neck cancer, the lateral tongue and oral pharyngeal mucosa, were examined in marijuana users and nonusers in order to determine if marijuana-specific differences occur in the makeup of these biofilms. It is well established that the oral biofilm on nonpathological mucosa is at least in part dependent on the nature of the mucosal cell character and the associated extracellular matrix^23,26,27^. This would suggest that monitoring of microbiota on oral surfaces provides a window into histology and biochemistry of the mucosa at that site, perhaps even before obvious histological changes occur^17^. Differences in bacteria at these cancer-prone sites may serve as a marker of marijuana usage associated change in oral mucosa that might precede cancer. The analysis provides insight on how daily or almost daily usage of combusted marijuana may be pathological at these sites.

## Results

### Selected subjects

There were twenty marijuana-user subjects, comprised of 17 males and 3 females. The control group, non-marijuana-users, consisted of 16 males and 3 females. The marijuana group subjects ranged from 18 to 49 years of age with mean age 25.7 and median age 24. That of the control group ranged from 18 to 58, with the mean age 27.3, and median age 25.5. None of the subjects had active caries or visible signs of more than mild gingival inflammation, nor did they have mucosal lesions at sample collection sites.

### Bacteria communities at oropharynx and lateral border of the tongue in marijuana users

**Figure 1:**
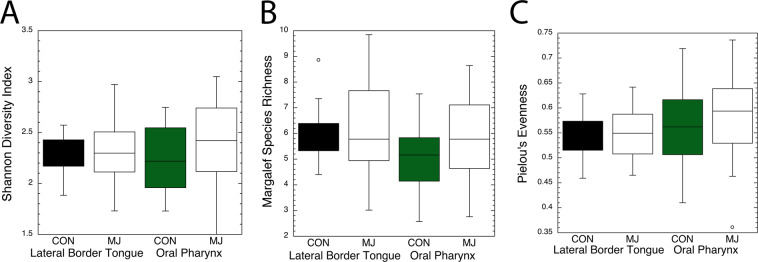
Boxplots of Shannon Diversity Index, Margalef Richness, and Pielou’s Evenness compare marijuana nonusers and marijuana (MJ) users for taxa identified at the two mucosal sites. The Student’s *t* test compared the significance of taxa differences for users and nonusers using each test. (**A)** Shannon, tongue t < 0.831 and oral pharynx t < 0.249. (**B)** Margalef, tongue t < 0.717 and oral pharynx t < 0.204. (**C)** Evenness, tongue t < 0.819 and oral pharynx, t < 0.507.

**Figure 2:**
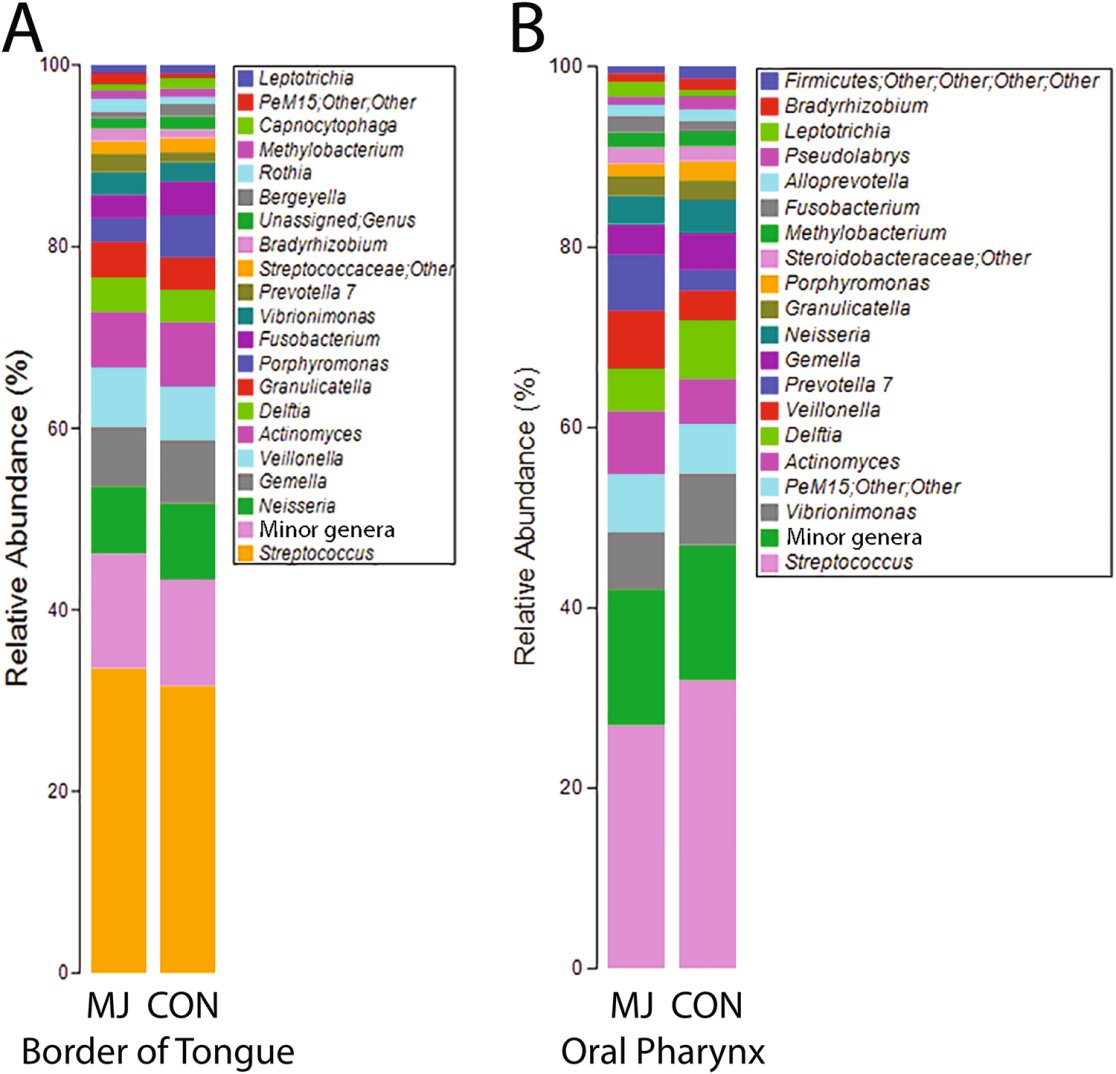
Bar plot of most common genera found at the two mucosal sites in marijuana users and control nonusers.

Profiles of bacteria communities from two cancer prone sites, the mucosal surface of the lateral border of the tongue and the oral pharynx, collected with a cotton swab were generated using 16s rDNA sequencing. This consisted of 1,950,345 raw reads for tongue and 2,053,618 raw reads from the oral pharynx. Bray-Curtis dissimilarity (non-phylogenetic) metric was used to perform analysis of similarity (ANOSIM), which indicated minimal overall differences between the two populations at both lateral border of the tongue (R = 0.033, p = 0.159) and the oral pharynx (R = 0.056, p = 0.075). Similarly, alpha diversity analysis revealed no differences in distributions in marijuana users and controls at either mucosal site, indicating that if there were differences in the populations they were limited to a minority of taxa (Fig. 1). The barplot in Fig. 2 reveals the 20 most common genera in marijuana users and nonusers at both sites.

### Examination of Individual taxa in the marijuana users and controls

16s rDNA based HOMD database taxonomic assignments revealed 67 genera detected at lateral border of the tongue and 65 at the oral pharynx. DESeq2 of these genera revealed potential differences at both sites between controls and marijuana users (Table 1), though with this test only those for tongue were significant after correction for multiple testing.

**Table 1:** DESeq2 differentiation of taxa on the genus level.

*Genus Lateral Border of the Tongue*	Mean Counts	Log2 Fold Change MJ/Con	p value	p adj
*Deftia*	632	4.03	3.24E-10	2.20E-08
*Leptothrix*	24	6.67	2.88E-05	9.81E-04
*Psuedomonas*	9.4	5.94	2.15E-03	4.88E-02
*Bosea*	135	5.86	3.15E-03	5.27E-02
*Lautropia*	181	2.55	3.88E-03	5.27E-02
*Rothia*	538	1.18	5.15E-03	5.84E-02
*Bergeyella*	393	−1.21	2.21E-02	2.15E-01
***Genus Oral Pharynx***	**Mean Counts**	**Log2 Fold Change MJ/Con**	**p value**	**p adj**
*Veillonella*	2227	0.906	5.23E-03	3.40E-01
*Prevotella*	2424	1.16	3.32E-02	4.57E-01
*Bergeyella*	118	−2.21	2.60E-02	4.57E-01
*Lysinibacillus*	26	−3.64	2.46E-02	4.57E-01
*Mogibacterium*	72	2.32	3.82E-02	4.57E-01
*Rumincoccaceae_[G-1]*	37	3.44	4.16E-02	4.57E-01

**Figure 3:**
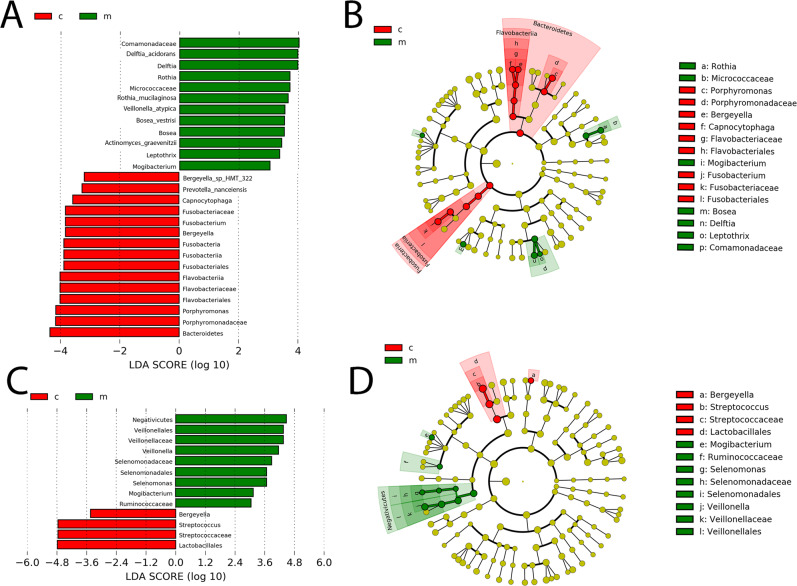
Taxa identified as distinct between marijuana, M, and control, C, groups at the two sites using LEfSe analysis. (**A**) LDA scores show significant differences in bacteria at the lateral border of the tongue in the marijuana and control nonusers. (**B)** Cladogram constructed shows the phylogenetic distribution of the differentially abundant taxa. (**C)** LDA scores show differences at the oral pharynx in the marijuana group and the controls. (**D)** Cladogram reveals phylogenetic distribution of differentially abundant taxa at the oral pharynx site in the marijuana and control groups.

Alternatively, to identify taxa that distinguish marijuana users from nonusers at both sites, the linear discriminant analysis (LDA) effect size (LEfSe) method was used to determine statistically differentially represented taxa while also taking account of biological consistency and performing effect size estimations (Fig. 3). Use of this approach revealed a number of species and genera that were potential markers of marijuana exposure. At the lateral border of the tongue, several species, *Rothia mucilaginosa*, *Delftia acidovorans*, *Veillonella atypica* and *Bosea vestrisii*, were higher in marijuana users, while three genera, *Fusoabcteria*, *Porphymonas*, and *Capnocytophaga*, were lower (Fig. 3C). A cladogram (Fig. 3B) was used to represent the predominant bacteria and the taxonomic relationship of the microbiota in both groups at the site. At the oral pharynx on the genus level, the abundance of *Veillonella* (*p* = 0.0001) was higher in marijuana users along with *Mogobacterium* and *Selenomonas*, while *Streptococcus* was lower (Fig. 3C,D).

### Measurement of bacteria found in commercially prepared marijuana at oral sites

16s rRNA gene sequenced datasets of operational taxonomic units (OTUs) from the tongue and oral pharynx sites were subjected to taxa assignment using the SILVA 132 comprehensive library of taxonomically known 16s rRNA genes^28^. The Silva 132 library includes 16s genes not included in the HOMD database of taxa that are very rarely found in saliva or other oral samples. Supplemental Figure S1 reveals of the 6 bacteria species shown earlier to be found reproducibly in commercially prepared marijuana, *Acinetobacter baumannii*, *Escherichia coli*, *Psuedomonas aeruginosa*, *Ralstonia pickettii*, *Salmonella enterica*, *and Stenotrophomonas maltophilia*, several were found at low levels at the lateral tongue and oral pharyngeal sites^29^. *Stenotrophomonas maltophilia* was discernible on the species level by 16s rDNA sequencing but found only at the tongue site in one marijuana subject and one nonuser. More subjects were positive based on 16s rDNA sequence for the genus *Stenotrophomonas* at this site but no difference in levels in marijuana versus negative control group was seen. *Ralstonia* and *Psuedomonas* were only differentiable at the genus level and based on 16s analysis, few samples had detectable levels, and again there was no difference whether the subject used marijuana or not. A species of *Pseudomonas*, *unidentified marine bacterioplankton*, was found in both marijuana and control subjects at both sites, lateral tongue and oral pharyngeal, but at similar low levels. Two additional bacterial species found in marijuana, *Acinetobacter bumanni* and *Escherichi Coli*, were not found in any subjects at the tested sites based on this analysis.

## Discussion

The results of this study suggest that daily or almost daily inhalation of marijuana in the past month correlates with differentially abundant taxa of the oral microbiome in samples taken from the lateral border of the tongue and from the oral pharynx. Both sites are distinct in regard to the marijuana-associated microbiome. Given that they are cancer-prone sites, one question arises: does marijuana use correlate with differences in the biofilm known to correlate with cancer? Several studies have catalogued, to varying degrees, biofilm differences in malignant HNSCC versus normal head and neck mucosa^18,20,21,30,31^. These tumor-associated taxa may be indicative of pathology, such as inflammation^18^, and not just malignancy when compared to healthy tissue. Several genera have been shown to be at higher levels with head and neck squamous cell carcinoma (HNSCC) and oral squamous cell carcinoma (OSCC), including *Fusobacterium, Capnocytophaga, Alloprevotella*, *Treponema*, *Campylobacter*, Selenomonas with other groups at lower levels: *Streptococcus*, *Veillonella*, *Lautropia*, *Actinomyces*, and *Rothia*. At the lateral tongue site, none of these differences were replicated in the marijuana users^18,21^. In fact, *Rothia* and *Lautropia* were higher, while *Capnocytophaga*, *Fusobacteria* and *Porphyromonas* were lower, with the latter thought to be mechanistically linked to OSCC^32^. In contrast, at the oral pharyngeal site, differences in marijuana users versus controls were more consistent with the cancer state. There were lower levels of *Streptococcus* and higher levels of *Selenomonas*, though *Veillonella* was higher^21,30,33^. It is quite possible that with marijuana use, we see these changes in bacteria levels that correspond with subtle changes in the mucosa that occur as normal tissue progresses toward pathology and ultimately SCC, but do not have a causative role in this process. Further research needs to be done to determine if marijuana shows these correlations in a larger population, whether marijuana is the causative factor for those changes in taxa, and the relationship of those bacteria with oral mucosal disease.

In conclusion, the lateral tongue site showed microbial changes with marijuana usage but these were inconsistent with cancer. Results with the oral pharynx were mixed, but overall more consistent with the malignant state. Interestingly, there has been research that found a 2.6 times more likely association of primary squamous cell carcinoma of the head and neck in marijuana users, once adjusting for cigarette smoking, alcohol use, and other risk factors^34^. However, this finding has not been consistent amongst all studies^15,16,35^ and a link between marijuana usage and OSCC is not well supported. There is more support for the observation that marijuana usage is associated epidemiologically with HPV-positive oral pharyngeal SCC^14,36^. This was corroborated in a second population^37^. In contrast, a negative correlation between marijuana usage and tongue SCC was seen. It is not clear if marijuana usage is associated with HPV infection^38,39^. The strong association of marijuana usage and oral pharynx SCC risk is consistent with what was observed in the marijuana users in regard to bacterial taxa at the oral pharynx site in this study.

The cross-sectional nature of this study makes it possible that any component of marijuana lifestyle may be responsible for the differences in oral pharynx and lateral border of the tongue biofilms. While we excluded tobacco users and controlled for age and gender, there are other potential confounders that may correlate with marijuana usage. However, if marijuana itself is directly responsible it is reasonable to assume reactive oxidative chemicals^2,3^, some produced in the burning process, can damage cellular and bacterial macromolecules and play a role in changing mucosa or bacteria so to alter biofilm microbes. The immunomodulatory activity of marijuana may also play a role in the makeup of the biofilm. Marijuana cannabinoids can alter apoptotic rates, cell proliferation and/or chemotaxis in T and B lymphocytes, and macrophage and dendritic cells^4–7^. Finally, marijuana, depending on the method of preparation, can contain a range of potentially pathogenic bacteria as live contaminants^29,40^. While we saw no evidence for elevated levels of marijuana product-associated bacteria contaminants at the two mucosal sites tested (Supplemental Fig. S1), oxidative and immunomodulatory effects of marijuana were not examined. Limitations of the study are the slight differences in of races and ethnicities in the two groups, with 8 Asians, 5 Caucasians, 4 Hispanics and 1 African American and three unknowns in the control group and 5 Asians, 9 Caucasions and 5 Hispanics and 1 African American in the marijuana group. (Supplemental Table 1). Differences in subgingival and salivary microbiota between Caucasian Americans and African Americans, Chinese Americans and Latin Americans have been noted^41^ while others have suggested the environment plays a larger role than genetics in oral microbiota^42,43^ It is also possible oral hygiene level may differ between the marijuana and control groups which can contribute to differences in mucosal microbiota. Finally, undetected periodontal disease, with minimal inflammation due to marijuana usage^4,5^, may be present in some patients and could contribute to taxa found at distal sites such as tongue and oral pharynx in those patients.

Both sites tested, oral pharynx and lateral border of the tongue, showed microbial differences with marijuana usage. This finding is consistent with the speculative model that mucosal biofilm constituents may provide a window to the state of mucosal health given the known effects of long-term marijuana usage on oral mucosa histology in some users. At the lateral border of the tongue the differences may not be carcinogenic, while for oral pharynx they may indeed be. Given the association of marijuana usage with HPV-induced SCC, and possibly HPV infections themselves, one model is that marijuana-related biofilm organism changes are manifestations of the same phenomenon – the immune state. Future experiments will test the effect of marijuana induction to a naive host and the effect on immune state and oxidative changes at these same sites. It will also be important to confirm the taxa differences seen at both mucosal sites in additional marijuana users and controls, and examine other variables so to better establish the relationship between marijuana usage and the taxa differences.

## Methods

### Study subjects

This pilot study consisted of 20 frequent marijuana users with 17 of these daily or almost daily users of marijuana in the past month (defined as using marijuana on 20 or more days in the past month)^44^. Exclusion criteria were systemic disease, overt oral disease such as active caries or moderate periodontitis, concurrent tobacco usage, usage of antibiotics in the last month^45^, usage of bactericidal mouthwash in the last 24 hours, and age less than 18 years. Controls who had never used marijuana nor tobacco were matched based on gender and age. Subjects who had clinically visible lesions at sites of sample collection were excluded. Participants were recruited from the dental clinics of the University of Illinois at Chicago College of Dentistry. All subjects provided written informed consent to participate in accordance with guidelines of the Office for the Protection of Research Subjects of the University of Illinois at Chicago, with formal approval of the study protocol by the Institutional Review Board 1 of the University of Illinois at Chicago.

### Sampling procedure

A cotton swab Fisherbrand 23-400-114 was used to collect samples from two intraoral sites (lateral border of the tongue and oral pharynx). Samples were placed into (TE) buffer solution (10 mmol Tris-HCl and 1 mmol EDTA) with a pH of 8.0 and frozen until DNA extraction could be completed.

### Characterization of microbial community structure

Genomic DNA was extracted from saliva samples using ZR Fungal/Bacterial DNA MiniPrep D6005 (Zymo Research Corp, Irvine, CA, USA) as recommended. Amplification reactions were performed targeting the V1-V3 variable regions of bacterial 16S ribosomal RNA (rRNA) genes using the primer sets 27F/534R, followed by a second amplification with barcoding and Illumina MiSeq sequencer as described earlier^46,47^. Library preparation and sequencing were performed at the University of Illinois at Chicago Sequencing Core^48^.

### Bioinformatics analysis

For taxa assignment and measurement, reverse sequences from the FASTQ files were analyzed using the software package QIIME2^49–51^. Sequences were trimmed if the average quality was lower than 20. As a result, the forward read sequences were truncated at 250 nt. and the reverse at 225 nt. Dada2-plugin in QIIME2 was used to sequence denoise and generate feature data and feature tables for the dataset^49,52^. It has earlier been shown that sequencing of V3 of 16S rDNA can be used to differentiate oral taxa when aligned to the HOMD annotated sequences^53^. Taxonomy assignment was done by classify-consensus-blast function with 98% match identity to the Human Oral Microbiome Database^54^. Of the 81 samples there were on average 32,746 reverse sequence reads generated per sample, ranging from 9884 to 46,125 reads. Samples with sequence reads below 9000 were eliminated prior to this analysis. For the alignment to the SILVA database of 16s rDNA sequences^28^ QIIME1.8 was used with merged paired reads of the V1-V3 region as described in the supplement and taxonomic assignment using the SILVA 132 database as described in the supplement^49,55,56^.

Alpha and beta diversity analysis were performed using Primer7^57^. This analysis included calculation of alpha diversity, Shannon’s diversity index of both species number and their distribution, Margalef’s of numbers, and Pielou’s of evenness of distribution^58–60^ and the significance of the differences between marijuana users and nonusers was derived using an unpaired Student’s *t* test. Beta diversity analysis was done using Bray Curtis dissimilarity (non-phylogentic) metric. ANOSIM) tests were performed to determine if microbial communities were significantly different between groups^47,57^. Differences in specific microbiota taxonomic abundance between the groups were tested using DESeq2 based on the negative binomial distribution^61^. Both this and the LEfSe analysis were performed after eliminating taxa that appeared in 15% or fewer subjects at >3 reads^62^.

## Supplementary information

Supplementary Methods, Table and Figure

## Data Availability

The sequencing data from this study is deposited in the Sequence Read Archive at NCBI.
