# Author Correction: Microbiomic differences at cancer-prone oral mucosa sites with marijuana usage

**DOI:** 10.1038/s41598-022-06597-7

**Published:** 2022-02-10

**Authors:** Taylor Newman, Laya P. Krishnan, Jessica Lee, Guy R. Adami

**Affiliations:** 1 Department of Periodontics, College of Dentistry, University of Illinois at Chicago, 801 South Paulina Street, Chicago, IL USA; 2 Department of Oral Medicine and Diagnostic Sciences, Center for Molecular Biology of Oral Diseases, College of Dentistry, University of Illinois at Chicago, 801 South Paulina Street, Chicago, IL USA

Correction to: *Scientific Reports* 10.1038/s41598-019-48768-z, published online 03 September 2019

The original version of this Article contained an error in the spelling of the author Taylor Newman which was incorrectly given as Taylor M. Newman. The original Article and accompanying Supplementary Information file have been corrected.

