# Supplementary material for: Microbiomic differences at cancer-prone oral mucosa sites with marijuana usage
Source: Sci Rep. 2019 Sep 3;9:12697. doi: 10.1038/s41598-019-48768-z (PMC6722050; doi:10.1038/s41598-019-48768-z)
Supplement: Supplementary file 1 — Supplementary Methods, Table and Figure [file 41598_2019_48768_MOESM1_ESM.pdf]

Microbiomic differences at cancer-prone oral mucosa sites with marijuana usage

Taylor M. Newman, Laya Krishnan, Jessica Lee, Guy R. Adami

Supplemental Methods

The QIIME v1.8 pipeline was used to perform the following processing steps to produce supplemental figure 1 [1]. Forward and reverse reads were merged using PEAR [2]. Ambiguous nucleotides (N) were trimmed from the ends and reads with internal ambiguous nucleotides were discarded. Reads were trimmed using a quality threshold of p = 0.01. Reads, after trimming, that wereless than 350 bp in length weredisscarded. Briefly, chimeric sequences were identified using the UCHIME algorithm as compared with the Silva 132 database [3]. Following this FASTA files for each sample were combined. Unique sequences were dereplicated from the combined sequences. Sequences with counts greater than 10were used as seed, or master, sequences for sequence clusters. Low abundance sequences were queried against the master sequences using USEARCH to find the master sequence with the highest percent identity with a cutoff of 98%. The counts for any sequence that matched a master sequence were then added to the counts for that cluster. Master sequences for each sequence cluster and any low abundance sequences that did not match a master were combined into a single file. Taxonomic annotations were assigned to each seed and independent low-abundance sequence using USEARCH and the Silva 132 reference database. Taxonomic and abunance data were merged into a single sequence table and summaries of absolute abundances of taxa were generated for all phyla, classes, orders, families, gener a, and species present in the dataset.

1 Caporaso, J. G. et al. QIIME allows analysis of high-throughput community sequencing data. Nature methods 7, 335-336, doi:10.1038/nmeth.f.303 (2010).

2 Zhang, J., Kobert, K., Flouri, T. & Stamatakis, A. PEAR: a fast and accurate Illumina Paired-End reAd mergeR. Bioinformatics 30, 614-620, doi:10.1093/bioinformatics/btt593 (2014).

3 Edgar, R. C. Search and clustering orders of magnitude faster than BLAST. Bioinformatics 26, 2460-2461, doi:10.1093/bioinformatics/btq461 (2010).

Supplemental Table 1
Pool of Subjects in Study

Subject	Age	Gender	MJ Usage	Ethnicity/Race	Subject	Age	Gender	MJ usage	Ethnicity/Race
MJ5	**42**	**M**	**Daily**	**Hispanic**	**MJ16**	**26**	**M**	**None**	**Asian**
MJ6	**18**	**M**	**Daily**	**Caucasian**	**MJ17**	**32**	**M**	**None**	**ND**
MJ7	**49**	**F**	**Daily**	**African American**	**MJ18**	**27**	**M**	**None**	**Caucasian**
MJ8	**25**	**M**	**Daily**	**Caucasian**	**MJ191**	**18**	**M**	**None**	**Hispanic**
MJ9	**25**	**M**	**Daily**	**Caucasian**	**MJ192**	**25**	**M**	**None**	**Hispanic**
MJ10	**23**	**M**	**Daily**	**Caucasian**	**MJ20**	**35**	**M**	**None**	**Hispanic**
MJ11	**26**	**M**	**4x/week**	**Caucasian**	**MJ21**	**20**	**M**	**None**	**Hispanic**
MJ12	**25**	**M**	**Daily**	**Caucasian**	**MJ22**	**22**	**M**	**None**	**Asian**
MJ13	**18**	**F**	**Daily**	**Hispanic**	**MJ23**	**24**	**M**	**None**	**Asian**
MJ14	**29**	**M**	**4x/week**	**Asian**	**MJ24**	**22**	**M**	**None**	**ND**
MJ15	**29**	**M**	**5x/week**	**Asian**	**MJ25**	**20**	**F**	**None**	**Asian**
MJ31	**23**	**M**	**Daily**	**Caucasian**	**MJ26**	**21**	**M**	**None**	**ND**
MJ32	**27**	**M**	**Daily**	**Hispanic**	**MJ27**	**27**	**M**	**None**	**Asian**
MJ33	**20**	**M**	**Daily**	**Asian**	**MJ28**	**49**	**F**	**None**	**Asian**
MJ34	**21**	**M**	**5x/week**	**Hispanic**	**MJ29**	**27**	**M**	**None**	**African American**
MJ35	**24**	**F**	**5x/week**	**Asian**	**MJ30**	**22**	**M**	**None**	**Asian**
MJ36	**24**	**M**	**Daily**	**Asian**	**MJ49**	**20**	**M**	**None**	**Asian**
MJ37	**23**	**M**	**Daily**	**Caucasian**	**MJ43**	**26**	**M**	**None**	**Caucasian**
MJ39	**23**	**M**	**2x/wk**	**Caucasian**	**MJ71**	**58**	**M**	**None**	**Caucasian**
MJ40	**23**	**M**	**Daily**	**Hispanic**	**MJ2311**	**26**	**F**	**None**	**Caucasian**

Supplemental Fig. S1

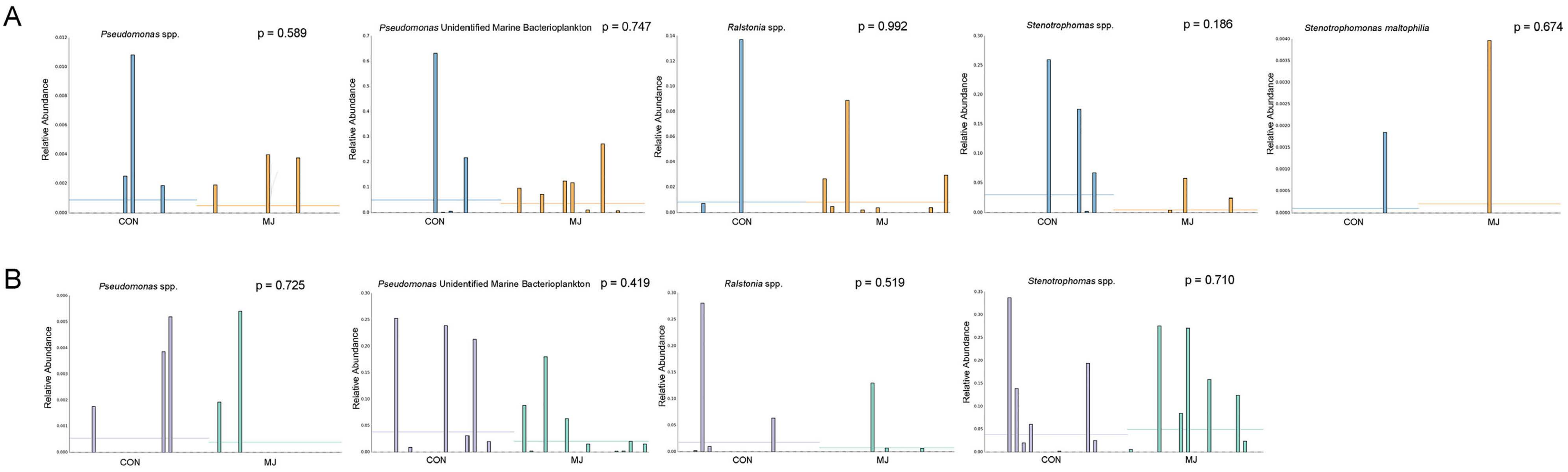

Supplemental Figure S1 Known bacterial marijuana contaminants, and related taxa, on the oral mucosa of marijuana users and controls. A. Relative levels of taxa in lateral border of the tongue samples are shown for each subject in the controls (CON) and the marijuana (MJ) groups. B Relative taxa in the oral pharynx samples are shown. Values for Student *t*-test comparisons are shown. White's nonparametric test showed a similar lack of statistically significant differences.
